# Exciton–phonon coupling in two-dimensional layered (BA)_2_PbI_4_ perovskite microplates†

**DOI:** 10.1039/d2ra06401d

**Published:** 2023-02-17

**Authors:** Yixiong Wang, Chenglin He, Qin Tan, Zilan Tang, Lanyu Huang, Liang Liu, Jiaocheng Yin, Ying Jiang, Xiaoxia Wang, Anlian Pan

**Affiliations:** a Key Laboratory for Micro-Nano Physics and Technology of Hunan Province, State Key Laboratory of Chemo/Biosensing and Chemometrics, College of Materials Science and Engineering, Hunan University Changsha Hunan 410082 China wangxiaoxia@hnu.edu.cn anlian.pan@hnu.edu.cn; b School of Physics and Electronics, Hunan University Changsha Hunan 410082 China

## Abstract

Two-dimensional layered (BA)_2_PbI_4_ (BA = C_4_H_9_NH_3_) perovskites are emerging as a new class of layered materials and show great potential in optoelectronic applications. Elucidating how exciton–phonon interaction affects the excitonic emission is of great importance for a better knowledge of their optoelectronic properties. In this letter, we synthesized high-quality (BA)_2_PbI_4_ microplates *via* solution methods, and dual-excitonic emission peaks (surface-emission and interior-emission) were detected from the as-grown samples at low temperatures. Furthermore, we determine the energies for the longitudinal optical phonon modes to be ∼27 and ∼18 meV, and the exciton–phonon coupling strengths to be ∼177 and ∼21 meV for the surface-emission and interior-emission bands, respectively. Compared to the interior-emission band, the stronger exciton–phonon interaction results in a considerable degree of spectral broadening and red-shift for the surface-emission with increasing temperature. In contrast, the (OA)_2_PbI_4_ (OA = C_8_H_17_NH_2_) microplates with longer alkyl chains between Pb–I layers, exhibit only one excitonic emission peak, as well as a large exciton–phonon coupling strength. Our work clarifies the influence of exciton–phonon coupling on the excitonic emission of (BA)_2_PbI_4_ microplates, and also suggests the intrinsic relationship between the exciton–phonon coupling and the length of organic carbon chain ligands.

## Abstract 2

The effect of exciton–phonon coupling on the excitonic emission of two-dimensional layered (BA)_2_PbI_4_ (BA = C_4_H_9_NH_3_) perovskites.

## Graphical abstract

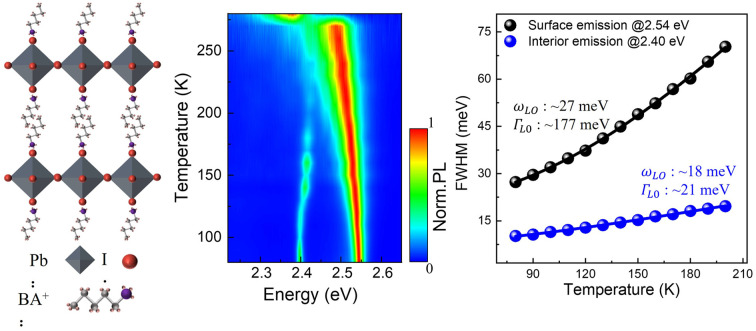

## Introduction

Two-dimensional (2D) perovskite materials are layered analogs of three-dimensional organic and inorganic hybrid halide perovskites, formed by larger alkyl ammonium cations partially or completely replacing commonly used methylammonium (MA) cations.^1–3^ Benefiting from advantages such as a simple preparation method,^4–6^ quantum well structure,^7,8^ and high exciton binding energy,^9–11^ these layered perovskites have been extensively studied and applied in light-emitting diodes,^12–14^ photodetectors,^15–17^ and solar cells.^18,19^ Among them, (BA)_2_PbI_4_ perovskites with butylammonium (BA^+^) organic cations shows novel photoluminescence (PL) characteristics due to its dynamic disorder and phase change originating from a relatively soft lattice structure.^20^ Meanwhile, crystallization conditions and the introduction of intrinsic defects and impurities will directly affect the photoelectric properties. In addition, (BA)_2_PbI_4_ with *n* = 1 is a simple and stable phase with strong excitonic emission in the visible region, which enables (BA)_2_PbI_4_ single crystals as a promising candidate to study the photophysical properties of 2D perovskites.^21^

Particularly, the dual-excitonic emission peak in (BA)_2_PbI_4_ crystalline has arisen in an intensive study. For instance, DeCrescent *et al.* demonstrate that the low-energy emission is due to magnetic dipole emission induced by p-like excitons, which self-trapped at a lower energy of 1s excitons than the primary electric dipole.^22^ Du *et al.* latest research suggest that this dual emission peak is induced by cation accumulation in (BA)_2_PbI_4_ bulk materials. Lattice residual strain originates from the decreased mobility of organic cations in the bulk or interior of the crystal.^23^ Recently, a demonstration of electronic interactions between the inorganic Pb–I layers was verified by intercalation of long-chain organic molecules in the layered perovskite single crystals at room temperature.^24^ These findings further validated prior's prediction that (BA)_2_PbI_4_ had a dual bandgap formed in the inside and the surface of the crystal. Generally, in the polar lattice of lead halide perovskites, it has become widely accepted that the Fröhlich interaction between exciton and optical phonons is crucial in describing its photonics properties. Subsequently, the dual emission peak of (BA)_2_PbI_4_ was also explained at a molecular level, revealing that a phonon replica of the excitonic state leads to the lower energy emission.^25^ It is not difficult to find that prior studies (BA)_2_PbI_4_ perovskites are most focused on the origin of the dual-emission peak, the specific influence of exciton–phonon interaction on the excitonic emission of (BA)_2_PbI_4_ perovskites remains elusive.

Here, single-crystal (BA)_2_PbI_4_ microplates were synthesized using a solution method. A typical excitonic recombination emission characteristic can be observed from the as-grown (BA)_2_PbI_4_ microplates at both the room temperature and low temperature. Furthermore, we examined the temperature dependence of the full width at half maximum (FWHM) and peak positions of the PL spectra. It is found that the exciton–phonon coupling of the surface emission is substantially stronger in comparison with that of the interior-emission, resulting in a considerable degree of the line width broadening for the surface-emission and a red-shift of bandgap with temperature increasing. In contrast, due to the longer alkyl chains between Pb–I layers in (OA)_2_PbI_4_ crystals, only one excitonic emission peak with strong exciton–phonon coupling is detected from (OA)_2_PbI_4_ microplates.

## Results

**Fig. 1:**
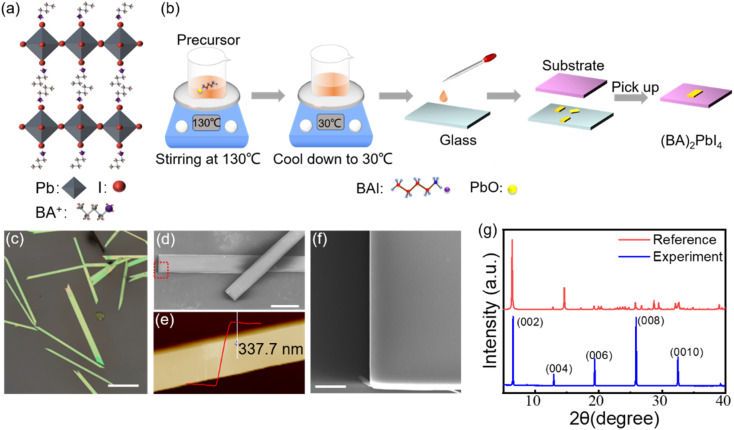
(a) Schematic diagram of the crystalline structure of (BA)_2_PbI_4_. (b) Schematic illustration of growth process of the (BA)_2_PbI_4_ microplates. (c) Optical image of the as-grown (BA)_2_PbI_4_ microplates. Scale bar is 200 μm. (d) SEM image of (BA)_2_PbI_4_. Scale bar is 40 μm. (e) AFM image of a representative (BA)_2_PbI_4_ microplate. (f) The enlarged SEM image of the marked region with a red rectangle in (d). Scale bar is 5 μm. (g) XRD spectra of the (BA)_2_PbI_4_ microplates along with the reference calculated from single crystal data.

The crystal structure of (BA)_2_PbI_4_ is shown in Fig. 1(a). The corner-shared [PbI_6_]^4−^ octahedra constitutes a layered semiconducting phase in this structure, which is separated by an insulating organic spacer BA^+^, generating a repeating quantum well structure. Fig. 1(b) shows the schematic diagram for the growth of (BA)_2_PbI_4_ microplates by using a solution strategy. The obtained (BA)_2_PbI_4_ microplates have a uniform morphology with tens of micrometers in lateral dimension and hundreds of micrometers in length, as illustrated in Fig. 1(c and d). Fig. 1(f) shows the enlarged SEM image of the marked region with a red rectangle in Fig. 1(d). It is found that the sample exhibits an excellent surface smoothness and a neat edge with a thickness of ∼337 nm [Fig. 1(e)], which indicates a high quality of the as-grown (BA)_2_PbI_4_ microplates. Additionally, the thickness of (BA)_2_PbI_4_ microplates can be precisely controlled by changing the temperature of the precursor (Fig. S1†). Fig. 1(g) shows the XRD of the (BA)_2_PbI_4_ microplates alongside a reference generated from single-crystal data, which shows a typical (00*l*) plane pattern of layered perovskites. Note that the position of the dominant diffraction peaks is almost similar when compared our experimental results with the reference data. Generally, the overall peak intensity depends more on the crystallinity of the sample and the signal of the experimental instrument.^26^ Herein, by comparing the peak position of the experimental and the reference of the XRD pattern, it is concluded that the crystal structure of the perovskites in this manuscript is consistent with the crystal structure in the reference. The high agreement between the experimental and calculated diffraction patterns indicates that the material is phase pure.^20^

**Fig. 2:**
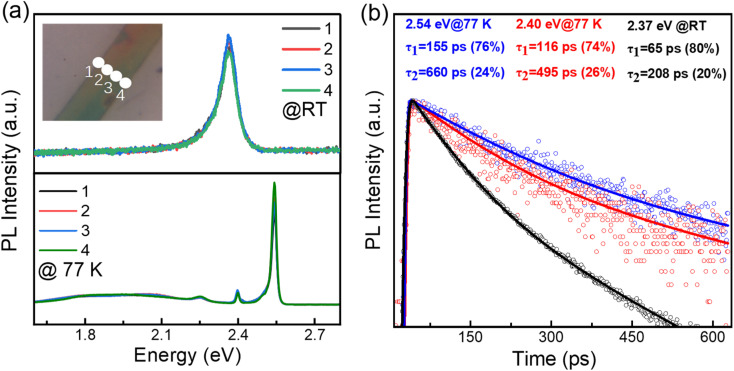
(a and b) Spatially-resolved PL emission spectra detected at room temperature (up panel) and at 77 K (bottom panel), locations are marked at inset of (a). (b) PL decay dynamics of the emission peaks of 2.54 eV and 2.40 eV at 77 K and 2.37 eV at room temperature, respectively.

An initial characterization of these single (BA)_2_PbI_4_ microplates is conducted *via* photoluminescence (PL) measurements, as shown in Fig. 2(a). The line-scan PL of a single microplate at room temperature show a uniform emission intensity at 2.37 eV and similar spectra profile, which further revealed a uniform crystalline quality of the as-grown sample. The UV-vis absorption spectra of the (BA)_2_PbI_4_ microplate at room temperature shows a strong absorbance that is associated with PL emission peak (Fig. S2†), which indicates that the PL peaks are not generated by defect states.^27^ While, at 77 K, the (BA)_2_PbI_4_ microplate exhibits prominent dual emission peaks located at 2.54 eV (488 nm) and 2.40 eV (517 nm) with a line-width of 22.56 meV and 11.04 meV, much narrower than that at room temperature. Note that the sample exhibits a broad emission ranging from ∼1.6 eV to ∼2.3 eV at lower temperatures. Such broad emission has been previously reported as self-trapped emission.^28–30^ The linear fitting of the emission peak intensity with the excitation power yields a slope of ∼1 both at room temperature and low temperature (Fig. S3†), which is consistent with an excitonic recombination process due to the large exciton binding energy of this type of 2D perovskites.^9,10,31^ In general, two-photon excitation show a deeper penetration depth into the microplate than one-photon excitation (400 nm), resulting in a high contribution of PL emission from the interior part of the sample. Fig. S4† shows the intensity of the emission peak located at 2.40 eV excited by 800 nm is much stronger than that observed with a 400 nm light excitation. To exclude that the photon might be in resonances with an intersubband transition when excited with 800 nm, the two-photon excitation process with another wavelength at 900 nm were studied (Fig. S5†). By examining the PL spectrum of (BA)_2_PbI_4_ with various thicknesses (Fig. S6†), we find the intensity of the low-energy emission peak increased as the thickness grew, further indicating the low-energy peak is associated with interior part emission of the sample and in consistent with the demonstration by Nag *et al.*^32^ Furthermore, the PL spectra of (BA)_2_PbI_4_ microplate at different focal points at 77 K were measured. As shown in the Fig. S7,† with the focal point changed (excitation depth changed), the intensity of the 2.40 eV emission has changed, which further demonstrates that the 2.40 eV emission originates from the “interior” part of the sample. Such that, we define the high energy emission at 2.54 eV and the low energy emission at 2.40 eV by using surface-emission (SE) and interior-emission (IE), respectively.

The time-resolved PL dynamics are further measured by streak camera and the results are shown in Fig. 2(b). At 77 K, the PL emissions show a biexponential decay feature with lifetimes of 155 ps (76%) and 662 ps (24%) for 2.54 eV emission, 116 ps (74%) and 495 ps (26%) for 2.40 eV emission, respectively. While, the room temperature PL lifetime of 65 ps (80%) and 208 ps (20%) is faster than that at low temperature, which could be ascribed to the inevitable increased nonradiative recombination channels at high temperature. These relatively short PL lifetimes further excluded the possibility of defect or trap-state emission, which usually have a lifetime as long as 100 ns.^33^

**Fig. 3:**
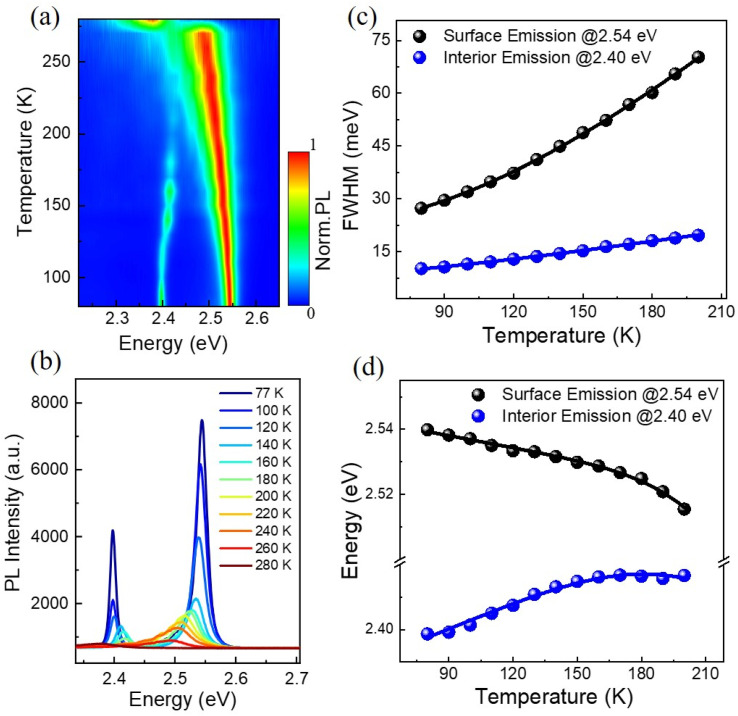
(a) The PL spectra of a 2D pseudo-color plot of the (BA)_2_PbI_4_ microplate at temperatures from 77 to 280 K. (b) Temperature-dependent PL spectrum of (BA)_2_PbI_4_ microplate. (c) The FWHM of the PL peak as a function of temperature extracted out from (b); dots: experimental data and line: the fitting curve. (d) The emission peak energy as a function of temperature extracted out from (b); dots: experimental data and line: the fitting curve.

Temperature-dependent photoluminescence spectra have long been used to investigate exciton–phonon coupling mechanisms in a variety of semiconductor materials.^34^Fig. 3(a) shows the 2D pseudo-color plot of temperature-dependent normalized photoluminescence spectra of the (BA)_2_PbI_4_ microplate. It is found that the IE band mainly emerged at low temperature range and vanished gradually when beyond ∼160 K, while the SE band keep existing with the temperature increasing and shift to ∼2.37 eV at 270 K is due to an phase transition,^35^ as also clearly shown in the temperature-dependent spectra [Fig. 3(b)]. The SE band is the low-temperature phase of (BA)_2_PbI_4_. It is noted that the SE band emission at ∼2.54 eV is the low-temperature phase of (BA)_2_PbI_4_. The phase transition occurs around ∼270 K and is related to the reconfiguration of BA molecule, which also affects the inorganic framework.^36^ During the phase transition process, the high-temperature domains within the crystal structure are created, which is frozen-out and manifested as an additional low-energy contribution around 2.4 eV,^37,38^ shown in Fig. 3(a). In addition, it is found that the 2.4 eV emission mainly emerged at low temperature range and vanished gradually when beyond ∼160 K [Fig. 3(b)], which is also consistent with the statement of the frozen-out of the high-temperature domains. There is no obvious defect-state associated broadband emission at the lower energy side throughout the temperature range, indicates a high crystalline quality of the (BA)_2_PbI_4_ microplate as compared to previously reported (BA)_2_PbI_4_ film or bulk crystals.^27,32^ In addition, the linewidth of both the SE and IE significantly narrower at low temperature, as to be expected due to a reduced contribution of exciton–phonon coupling stemming from diminishing phonon populations.^39^ Remarkably, the degree of this narrowing is different (∼10 meV for the IE and 43 meV for the SE) after we extracted the temperature dependence of the FWHM of the two emission peaks, as shown in Fig. 3(c).

In general, the primary contribution to the temperature-dependent linewidth broadening is determined to be the Fröhlich interaction of excitons with longitudinal-optical phonons (LO), while acoustic phonons or impurity scattering usually playing a diminished role. To further quantify the Fröhlich coupling strength of this dual-excitonic emission, we employ the following model to fitting their temperature dependence line width broadening:^34^1
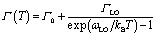
Here, *Γ*_0_ represents the temperature-independence inhomogeneous broadening term, and it is related to the scattering processes associated with disorder and imperfections of the lattice. The second term represents the homogeneous broadening term which originates from the Fröhlich coupling with the LO phonons. *k*_B_ is the Boltzmann constant. *Γ*_LO_ and *ω*_LO_ correspond to the Fröhlich coupling strength and longitudinal optical phonon energy, respectively. These parameters are fitted by eqn (1): *Γ*_0_ ∼ 24 meV, *ω*_LO_ ∼ 27 meV, *Γ*_LO_ ∼ 177 meV for SE and *Γ*_0_ ∼ 9.6 meV, *ω*_LO_ ∼ 18 meV, *Γ*_LO_ ∼ 21 meV for IE. The fitted LO phonon energy is comparable to the previous measured 2D halide perovskite nanoplatelets counterparts, but a little bit larger than that of 3D perovskites (FAPbI_3_), which further demonstrate the soft lattice features of the as-grown 2D (BA)_2_PbI_4_ microplates.^40–42^ Additionally, the LO phonon energy for surface emission ∼27 meV is slightly larger than that of interior-emission ∼18 meV. Notably, the Fröhlich coupling strength ∼177 meV for the SE is much higher than that of the IE (∼21 meV). Such a high Fröhlich coupling strength, *Γ*_LO_, at the surface region of the sample explains why the SE band exhibits a larger degree of total thermal broadening compared with the IE band.

The temperature dependence of the peak position is analyzed as a further step in getting more knowledge about the exciton–phonon interaction of the dual-emission band. It is noted that the surface emission redshifts as the temperature rises, but the interior emission follows an opposite trend [Fig. 3(b)], which differs from previously reported dual-peak emission of bulk (BA)_2_PbI_4_ perovskites.^32^ For perovskite materials systems, when the temperature rises, thermal-induced lattice expansion reduces the valence bandwidth and thus increases the bandgap (*E*_g_).^43^ Simultaneously, the electron–phonon interaction will be enhanced since the phonon density growing with temperature, enabling a red-shift of emission energy. As a result, the variation of *E*_g_ with temperature should be a competitive effect between lattice thermal expansion (TE) and exciton–phonon (EP) coupling. By assuming a linear relationship between lattice constant and temperature bandgap, the temperature-dependence bandgap variation of the dual-excitonic emission can be fitted by the following expression:^44^2
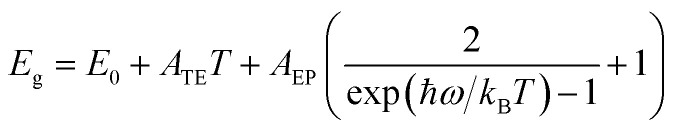
where *E*_0_ is the bandgap at 0 K, *A*_TE_ and *A*_EP_ are the TE and EP interaction strength, ℏ*ω* is the optical phonon energy, and *k*_B_ is the is the Boltzmann constant. Since we are particularly interested in the *A*_TE_ and *A*_EP_, we fix the energy of the phonons responsible for the shift ℏ*ω* to the LO phonon energy *ω*_LO_ derived from Fig. 3(c). Fig. 3(d) shows temperature dependence of the dual-emission peak energies fitted by eqn (2). The following parameters are obtained: *A*_TE_ ∼ 0.07 meV K^−1^, *A*_EP_ ∼ −60 meV for SE and *A*_TE_ ∼ 0.4 meV K^−1^, *A*_EP_ ∼ −28 meV for IE. According to the calculated parameters, *A*_TE_, the influence on bandgap shift from thermal expansion of the lattice is only 0.07 meV K−1, which is insufficient to offset the changes caused by strong exciton–phonon coupling, resulting in a progressive redshift of the SE peak energy. In contrast, the weight of *A*_TE_ is 0.4 meV K^−1^ for the IE, implying that lattice thermal expansion suppresses the exciton–phonon coupling effect and dominates bandgap shifting, as seen by the virtually linear blueshift of the peak energy.

As prior report demonstrate that lower-energy emission is induced by the organic cation stacking effect in the interior part.^24^ Such that the above results and discussions might suggests that longer alkyl chains of surface regime induce a larger local lattice distortion in the Pb–I layers compared to the shorter chains of the interior part, resulting a stronger exciton–phonon interaction in the surface emission (BA)_2_PbI_4_ perovskites. Additionally, as the temperature increase, lattice expansion will counteract this stacking effect, thus the interior emission band disappeared.

**Fig. 4:**
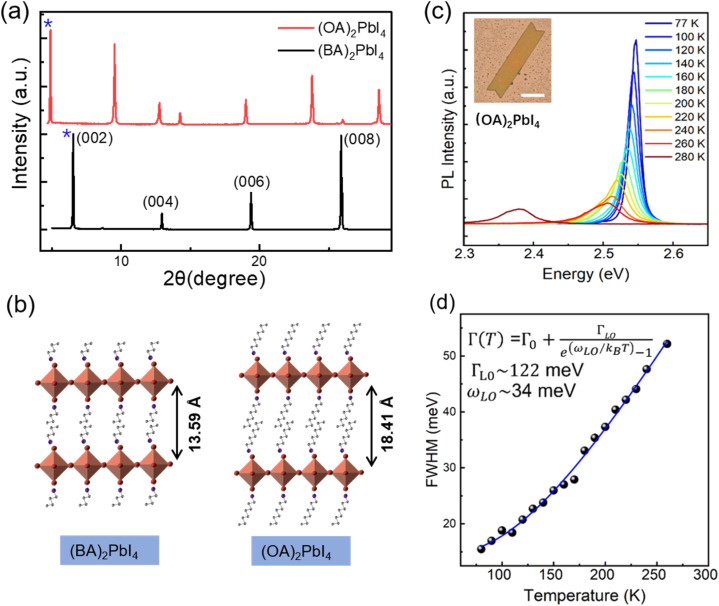
(a) XRD spectra of the (BA)_2_PbI_4_ and (OA)_2_PbI_4_ microplates. (b) Schematic of crystal structures of (BA)_2_PbI_4_ and (OA)_2_PbI_4_ showing interlayer distance obtained from XRD patterns shown in (a). (c) Temperature-dependent PL spectra of (OA)_2_PbI_4_ microplate, inset is the optical image of single (OA)_2_PbI_4_ microplate, the scale bar is 50 μm. (d) The FWHM of the PL peak as a function of temperature extracted out from (c); dots: experimental data and line: the fitting curve.

In order to probe the correlation between the exciton–phonon coupling and the alkyl chains length of the organic cations, we further fabricate (OA)_2_PbI_4_ microplates and studied the temperature dependent PL. The XRD patterns in Fig. 4(a) show the peaks are shifted toward the lower 2*θ* values from (BA)_2_PbI_4_ to (OA)_2_PbI_4_. In order to accurately analyze the Pb–I inorganic layer spacing of (BA)_2_PbI_4_ and (OA)_2_PbI_4_, the small angle XRD patterns were further obtained (Fig. S8†). According to their small angle XRD data, the Pb–I inorganic layer spacing is calculated to be 13.59 Å for (BA)_2_PbI_4_ and 18.41 Å for (OA)_2_PbI_4_ by Bragg's law, respectively.^45^ As the lattice structure depicted in Fig. 4(b), the difference between (OA)_2_PbI_4_ and (BA)_2_PbI_4_ mainly lies in alkyl chain length. While BA^+^ and OA^+^ are both linear alkyl chains within the same group, which has little effect on their emission wavelength with the main peak located at 2.54 eV at 77 K, as can be seen in Fig. 4(c). However, unlike the dual-excitonic peak detected from (BA)_2_PbI_4_, (OA)_2_PbI_4_ only show one emission peak within the temperature window we examined and just as the spectra profile of surface emission for (BA)_2_PbI_4_. In addition, by fitting the temperature-dependence FWHM of the PL spectra, we obtained the LO phonon energy and the exciton–phonon coupling strength of *ω*_LO_ ∼ 34 meV and *Γ*_LO_ ∼ 122 meV, respectively [Fig. 4(d)]. The obtained LO phonon energy is a little bit large than the surface emission of (BA)_2_PbI_4_, which further indicates longer alkyl chains is responsible for a large local lattice distortion and higher phonon energy. In addition, the strong exciton–phonon interaction similar as detected from the surface emission of (BA)_2_PbI_4_. These observations demonstrate the intrinsic correlation between the exciton–phonon coupling and the length of alkyl chain of two-dimensional layered hybrid perovskites, as well as the origin of lower energy emission.

## Conclusions

In conclusion, we have experimentally studied the influence of exciton–phonon coupling on the excitonic emission in (BA)_2_PbI_4_. The excitation power-dependent PL both in room temperature and 77 K indicate a typical excitonic recombination characteristic of the as-grown (BA)_2_PbI_4_ microplates. According to the temperature dependent PL, the exciton–phonon interaction strength of the surface-emission is much stronger than that of the interior-emission, resulting a redshift of the emission spectrum and larger bandwidth broadening. In contrast, only single emission peak was detected from (OA)_2_PbI_4_ microplates and distance between Pb–I inorganic layers of (OA)_2_PbI_4_ is larger than that of (BA)_2_PbI_4_, indicating the length of alkyl chains affects the lattice distortions of Pb–I layers and the exciton–phonon coupling in 2D layered perovskites. Our findings will be beneficial for the optimization of light sources based on 2D layered halide perovskites.

## Conflicts of interest

There are no conflicts to declare.

## Supplementary Material

RA-013-D2RA06401D-s001
