# Supplementary material for: Exciton–phonon coupling in two-dimensional layered (BA)2PbI4 perovskite microplates
Source: RSC Adv. 2023 Feb 17;13(9):5893–9. doi: 10.1039/d2ra06401d (PMC9936372; doi:10.1039/d2ra06401d)
Supplement: RA-013-D2RA06401D-s001 [file RA-013-D2RA06401D-s001.pdf]

Electronic Supplementary Material (ESI) for RSC Advances.
This journal is © The Royal Society of Chemistry 2023

SUPPLEMENTARY MATERIAL

Exciton-phonon coupling in two-dimensional layered $(BA)_2PbI_4$ perovskites microplates

Yixiong Wang,[‡] Chenglin He,[†] Qin Tan,[†] Zilan Tang,[†] Lanyu Huang,[‡] Liang Liu,[†] Jiaocheng Yin,[†] Ying Jiang,[‡] Xiaoxia Wang,[†] Anlian Pan[†]

†Key Laboratory for Micro-Nano Physics and Technology of Hunan Province, State Key Laboratory of Chemo/Biosensing and Chemometriscs and College of Materials Science and Engineering, Hunan University, Changsha, Hunan 410082, China

‡School of Physics and Electronics, Hunan University, Changsha, Hunan 410082, China

Synthesis and Characterization

Synthesis of $(BA)_2PbI_4$ perovskite microplates. All chemicals were purchased from p-OLED and used as received unless otherwise stated. First, the perovskite precursor was prepared: 0.25M PbO was dissolved in the acid mixture of HI and H_3PO_2 (10: 1 vol/vol), and stirred at 130°C for 5 minutes, yellow solution was obtained. Then, 0.25M BAI was added to the precursor and stirred for 3h. Subsequently, the stirring was stopped, the final precursor solution was naturally cooled to 30°C and the precursor supernatant was kept at a constant temperature of 30°C. 10 μL perovskite precursor solution was dropped on the glass slide. Then gently place the substrate on the droplet and lift it up. $(BA)_2PbI_4$ microplates were prepared on the substrate. We can grow the $(BA)_2PbI_4$ microplates of different thicknesses by controlling the temperature of the precursor solution. (Fig. S1).

Synthesis of $(OA)_2PbI_4$ perovskite microplates. All chemicals were purchased from p-OLED and used as received unless otherwise stated. First, the perovskite precursor

was prepared: 0.028M PbO was dissolved in the acid mixture of HI and H_3PO_2 (10: 1 vol/vol), and stirred at 130°C for 5 minutes, yellow solution was obtained. Then, 0.028M OAI was added to the precursor and stirred for 3h. Subsequently, the stirring was stopped, the final precursor solution was naturally cooled to 30°C and the precursor supernatant was kept at a constant temperature of 30°C. 10 μL perovskite precursor solution was dropped on the glass slide. Then gently place the substrate on the droplet and lift it up. $(OA)_2PbI_4$ microplates were prepared on the substrate.

Optical Measurements. μ-PL and absorption measurements were carried out on a commercial confocal μ-PL system (WITec, alpha-300). A mode-locked pulsed laser at 400 nm and 800 nm were used to excite PL of the $(BA)_2PbI_4$ and $(OA)_2PbI_4$, respectively. For the time-resolved PL spectroscopy, frequency-doubled output (400 nm) from a 800 nm mode-locked pulsed laser (Spectra-Physics) was employed as an excitation source, with a pulse width of 80 fs and frequency of 80 MHz, and the PL decay spectra were detected by a streak camera system (Hamamatsu Inc.)

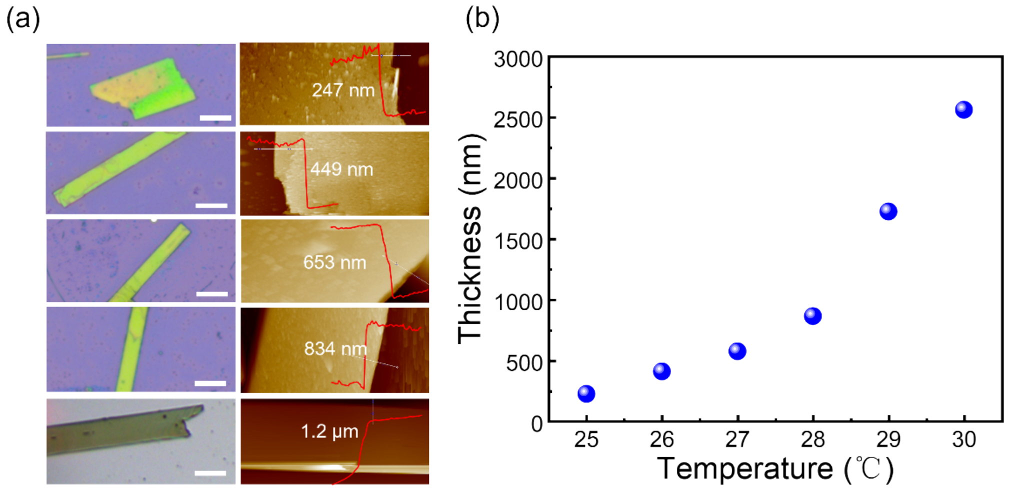

FIG S1. (a) Optical and AFM images of different thickness $(BA)_2PbI_4$ microplate. (b) Temperature-dependent thickness curve. The scale bar is 20 μm.

The thickness of $(BA)_2PbI_4$ microplates can be modulated by changing the temperature of the precursor. $(BA)_2PbI_4$ microplates of different thicknesses can be obtained by precursors at different temperatures. As shown in Fig. S1(b), the thickness of 100-200 nm microplates can be obtained at 25°C, and μm grade microplates can be obtained at about 30°C.

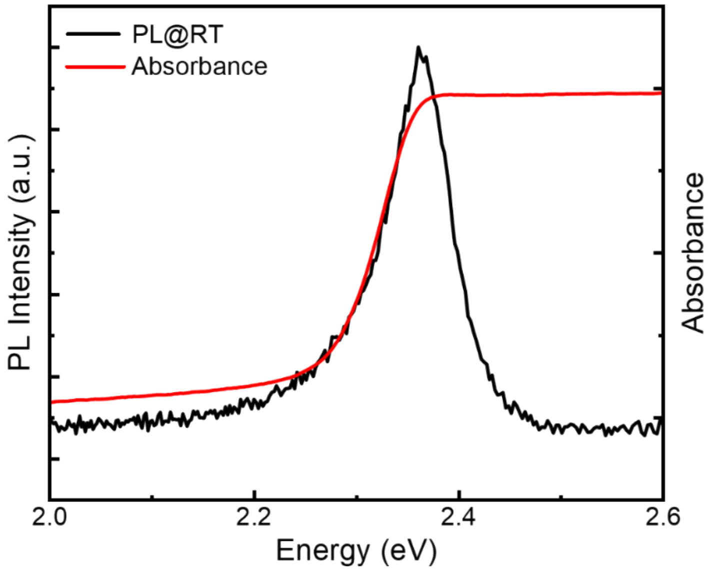

FIG S2. The absorption and PL spectra of $(BA)_2PbI_4$ microplates.

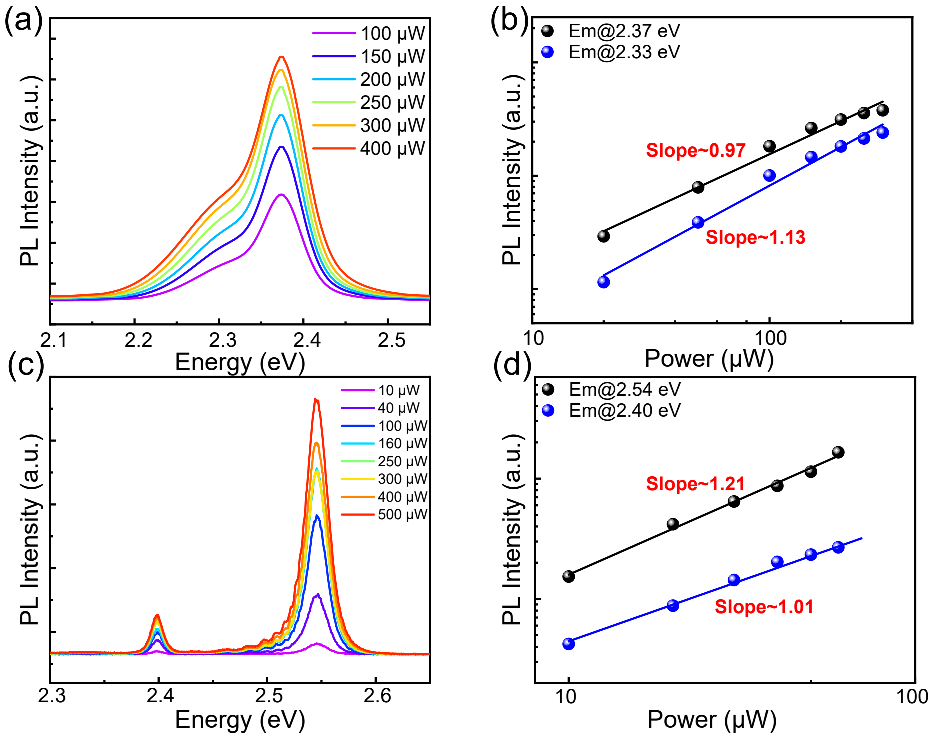

FIG S3. (a) The excitation power-dependent PL spectrum of $(BA)_2PbI_4$ microplate at room temperature. (b)The linear fitting of the emission peak intensity at room temperature. (c) The excitation power-dependent PL spectrum of $(BA)_2PbI_4$ microplate at 77 K. (d) The linear fitting of the two-emission peak intensity at 77 K.

At room temperature and low temperature, the intensity of the emission peaks increases as the excitation power increases. As can be seen from Fig S3a, for some of the as-grown samples, we observed a shoulder peak from the lower energy side, which might be ascribed to the defects-related emission or photon recycling[1, 2]. The linear fitting of the emission peak intensity with the excitation power yields a slope of ~0.97 and ~1.13, ~1.21 and ~1.01 for room temperature and low temperature, respectively (Fig. S3b and d). which is consistent with an excitonic recombination process due to the large exciton binding energy of this type of 2D perovskites[3].

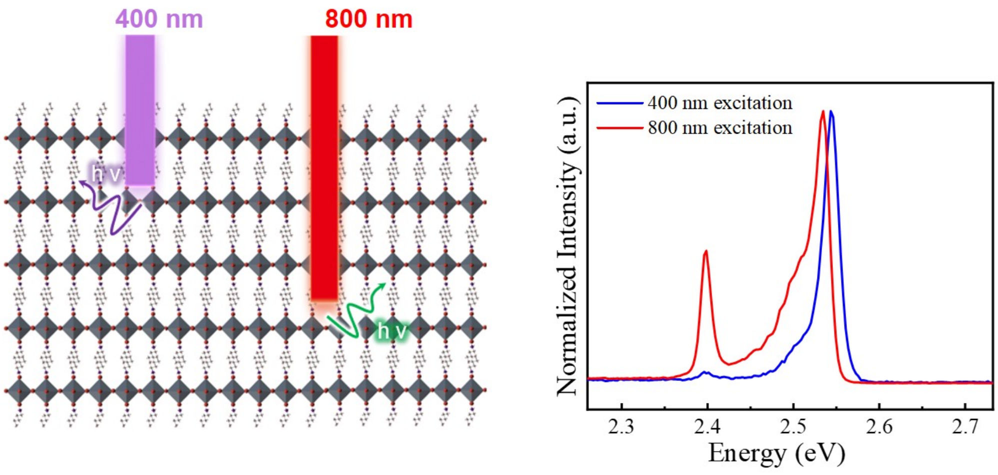

FIG S4. (a) The schematic illustration of the difference in penetration depth with 400 nm and 800 nm light. (b) PL spectra of $(BA)_2PbI_4$ microplates were measured with different excitation wavelength at 77 K.

The penetration depth δ depends strongly on the absorption coefficient α, according to the formula: $\delta = 1/2\alpha$, where $\alpha = 4\pi k/\lambda$, k is the extinction coefficient and λ is the wavelength[4]. According to the equation, two-photon excitation has a longer penetration depth compared with one-photon excitation. Therefore, under one-photon and two-photon excitation, most of the photoluminescence is generated from the surface and bulk regions, respectively[5]. For the differences between surface and bulk regions, such as band structure and carrier mobility, the two-photon excited charge carriers are located inside of the materials and show different carrier dynamics and emission properties. As shown in Fig. S4, the intensity of the emission peak located at 2.40 eV excited by 800 nm is much stronger than that observed with a 400 nm light excitation.

The 2.54 eV emission will be reabsorbed before being acquired by the detector while propagation from the initial emitting spot toward the surface, which will result in the PL redshift as a function of the distance between the excitation and detection point.

As we discussed, the penetration depth with 800 nm excitation is deeper than 400 nm. Therefore, as shown in Fig. S4. The peak position of 2.54 eV redshifts as the depth increases, might mainly ascribe to the increasing number of photon recycling for the detected PL[1].

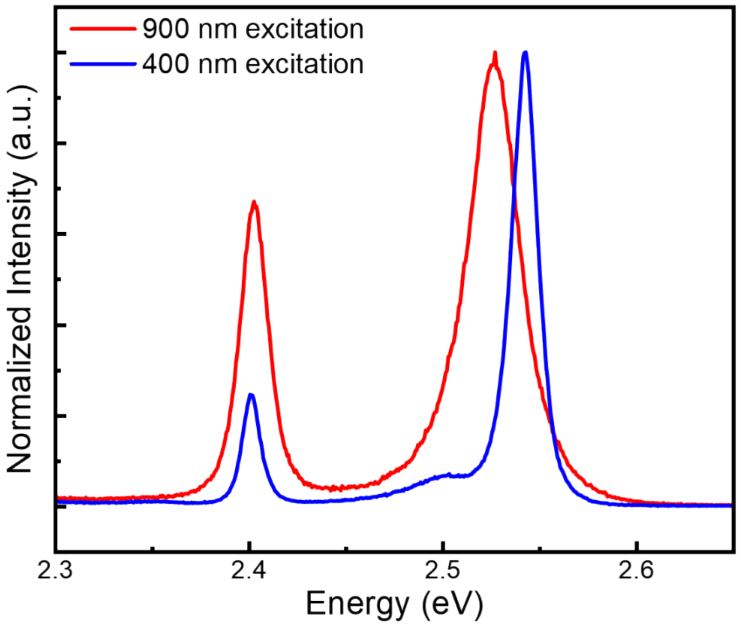

FIG S5. PL spectra of $(BA)_2PbI_4$ microplates were measured with different excitation wavelength (400 and 900 nm) at 77 K.

To excluded that the photon might be in resonances with an intersubband transition when excited with 800 nm, the two-photon excitation process with another wavelength at 900 nm were studied. It is found that the emission peak located at 2.40 eV excited by 900 nm is much stronger than that observed with a 400 nm excitation, which is consistent with the results of excitation pairs (400 and 800 nm). Therefore, the effect mentioned above can be excluded and further explained that the 2.40 eV emission is from the interior of the sample.

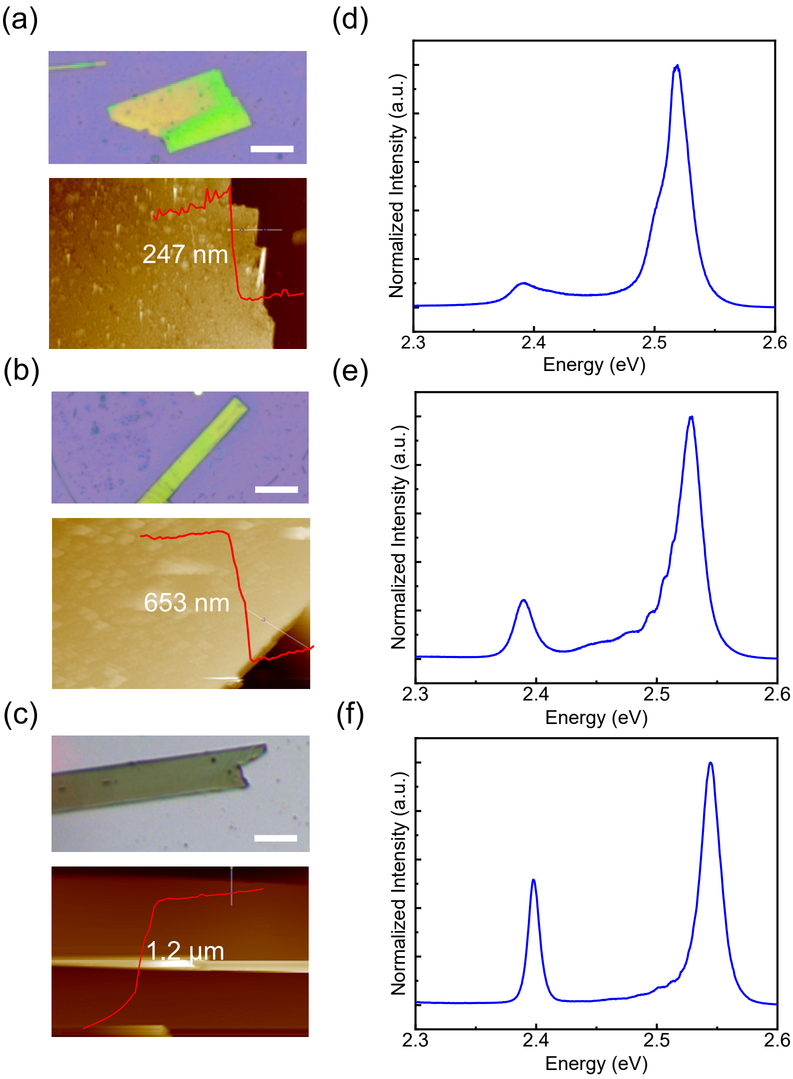

FIG S6. (a), (b) and (c) Optical and AFM images of different $(BA)_2PbI_4$ microplates. (d), (e) and (f) The corresponding PL spectrum of $(BA)_2PbI_4$ microplate at 77 K. Scale bar is 20 μm.

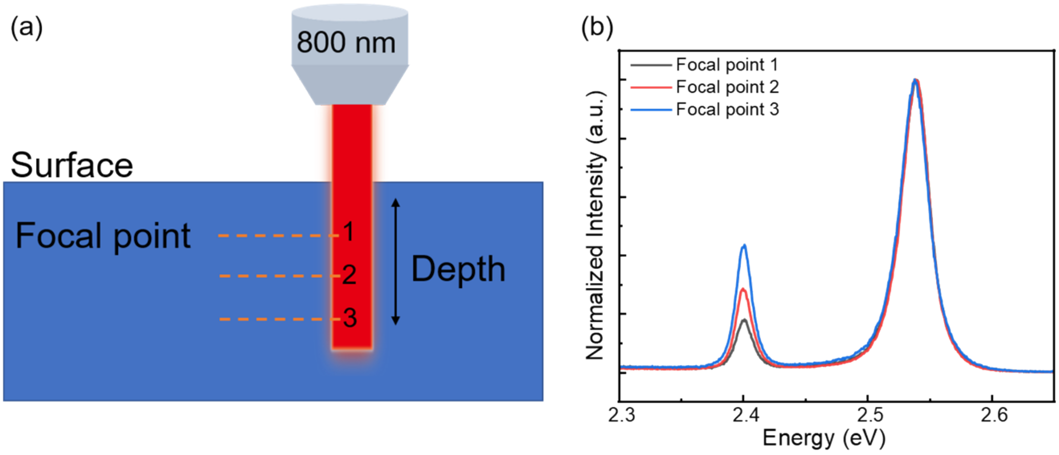

FIG S7. (a) The schematic illustration of the different focal point with 800 nm. (b) PL spectra of $(BA)_2PbI_4$ microplates were measured at different focal points at 77K.

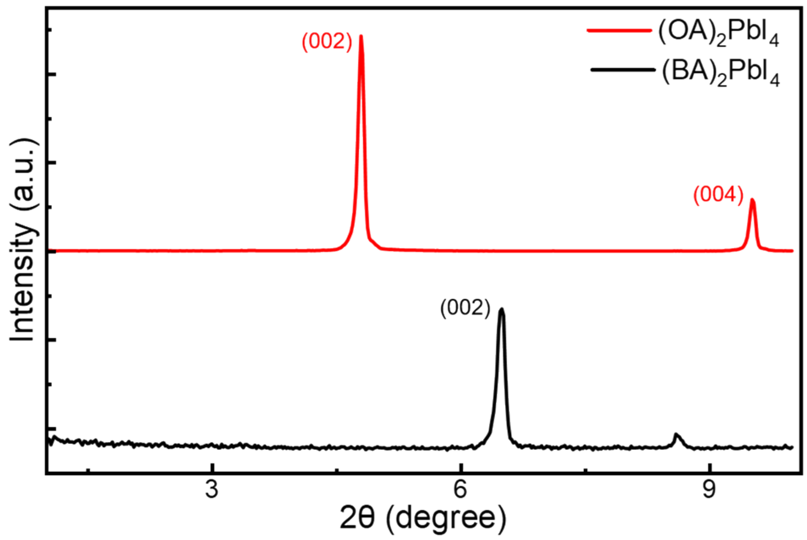

FIG S8. Small angle XRD spectra of the $(BA)_2PbI_4$ and $(OA)_2PbI_4$ microplates.

References:

1. Z. Gan, X. Wen, W. Chen, C. Zhou, S. Yang, G. Cao, K. P. Ghiggino, H. Zhang and B. Jia, *Adv. Energy Mater.*, 2019, **9**, 1900185.
2. J.-C. Blancon, J. Even, C. C. Stoumpos, M. G. Kanatzidis and A. D. Mohite, *Nature Nanotechnology*, 2020, **15**, 969-985.
3. J.-C. Blancon, H. Tsai, W. Nie, C. C. Stoumpos, L. Pedesseau, C. Katan, M. Kepenekian, C. M. M. Soe, K. Appavoo, M. Y. Sfeir, S. Tretiak, P. M. Ajayan, M. G. Kanatzidis, J. Even, J. J. Crochet and A. D. Mohite, *Science*, 2017, **355**, 1288-1292.
4. K. Piskorski, M. Guziewicz, M. Wzorek and L. Dobrzanski, *AIP Adv.*, 2020, **10**, 055315.
5. J. Chen, W. Zhang and T. n. Pullerits, *Mater. Horiz.*, 2022, **9**, 2255.
